# Landscape of tumor and immune system cells-derived exosomes in lung cancer: mediators of antitumor immunity regulation

**DOI:** 10.3389/fimmu.2023.1279495

**Published:** 2023-10-17

**Authors:** Alejandro Castillo-Peña, Sonia Molina-Pinelo

**Affiliations:** ^1^Institute of Biomedicine of Seville (IBiS), HUVR, CSIC, University of Seville, Seville, Spain; ^2^Spanish Center for Biomedical Research Network in Oncology (CIBERONC), Madrid, Spain

**Keywords:** exosome, lung cancer, immune cell, immunotherapy, tumor microenvironment

## Abstract

The immune system plays a critical role in cancer, including lung cancer, which is the leading cause of cancer-related deaths worldwide. Immunotherapy, particularly immune checkpoint blockade, has revolutionized the treatment of lung cancer, but a large subset of patients either do not respond or develop resistance. Exosomes, essential mediators of cell-to-cell communication, exert a profound influence on the tumor microenvironment and the interplay between cancer and the immune system. This review focuses on the role of tumor-derived exosomes and immune cells-derived exosomes in the crosstalk between these cell types, influencing the initiation and progression of lung cancer. Depending on their cell of origin and microenvironment, exosomes can contain immunosuppressive or immunostimulatory molecules that can either promote or inhibit tumor growth, thus playing a dual role in the disease. Furthermore, the use of exosomes in lung cancer immunotherapy is discussed. Their potential applications as cell-free vaccines and drug delivery systems make them an attractive option for lung cancer treatment. Additionally, exosomal proteins and RNAs emerge as promising biomarkers that could be employed for the prediction, diagnosis, prognosis and monitoring of the disease. In summary, this review assesses the relationship between exosomes, lung cancer, and the immune system, shedding light on their potential clinical applications and future perspectives.

## Introduction

Lung cancer has a significant impact worldwide: it is the leading cause of cancer-related deaths and the second most commonly diagnosed type of cancer (1). Most lung cancers are diagnosed at an advanced stage, which contributes to the poor survival rate of the disease. Environmental factors play an important role, as tobacco smoke is the leading cause of lung cancer, both through primary and second-hand exposure (2). Lung cancer is classified into two main groups, based on morphology and immunohistochemistry, small-cell carcinoma (SCLC) and non-small-cell carcinoma (NSCLC). SCLC has a worse prognosis, although NSCLC is the predominant type, representing 83% of diagnosed cases, and is subdivided into adenocarcinoma (ADC), squamous cell carcinoma (SqCC) and other minority subgroup, such as large-cell carcinoma (LCLC) (3). ADC is the most common type of NSCLC, accounting for over 40% of all lung cancer cases. It is defined as a malignant epithelial tumor exhibiting glandular differentiation, which can produce mucin. ADC presents, in general, a peripheral location in the lung. In the case of SqCC, it represents about 20% of lung cancer cases and is usually located in a central region. SqCC is defined as a malignant epithelial tumor that shows keratinization and/or intercellular bridges (4). Smoke inhalation has a more significant influence on SqCC, which was the most common subtype of lung cancer before 1998-2002, than on ADC. This shift seems to be related to changes in smoking behavior (5). NSCLC englobes a heterogeneous group of subtypes widely divergent from each other, as can be observed comparing ADC and SqCC (6).

The immune system plays a fundamental role in cancer (7). Lung cancer is no exception, and immunotherapy has revolutionized its treatment in the last decade, despite being considered a non-immunogenic disease in the past (8). In particular, immune checkpoint blockade has transformed the landscape of cancer immunotherapy, demonstrating unprecedented clinical efficacy in more than 15 cancer types, including NSCLC, through the use of programmed death 1 (PD1) and programmed death 1 ligand (PDL1). Moreover, immune checkpoint inhibitors (ICI) have recently been established as a first-line treatment for SCLC in combination with chemotherapy, although with modest clinical impact (9). Tumor cells habitually present immune evasion mechanisms of diverse nature and specifics that suppress the immune response. Comprehending these mechanisms and developing treatments such as the one mentioned above, which make it possible to overcome them with significant efficacy and thus enhance the antitumor immune response, is the backbone of immunotherapy.

Unfortunately, only a subset of patients shows benefits from immunotherapy. The development of innate or acquired resistance, in addition to late diagnosis in lung cancer, highlights the need for new biomarkers that allow identifying tumor characteristics and optimal therapy. Therefore, interest in liquid biopsy has been raised in recent years (10). For clinical diagnosis, liquid biopsy has some advantages over solid biopsy: there are easy access and minimally invasive samples, which show better tumor heterogeneity. Furthermore, liquid biopsy can be used for real-time tumor monitoring (11). Any biological fluid containing tumor material can be used, such as blood, pleural fluid or saliva, among others (12). The main biomarkers characterized in liquid biopsy samples are circulating tumor DNA, circulating tumor cells and exosomes (11, 12) The main topic of this review is the role of exosomes in lung cancer immune response.

## General characteristics of exosomes

Exosomes are single-membrane secreted vesicles, between 30 and 200 nanometers in diameter, with the same topology as the cell. Exosomes play a myriad of biological roles, including the remodeling of the extracellular matrix (ECM), and exosome-mediated signaling and molecular transfer between cells (13). All types of cells release exosomes and other extracellular vesicles (EVs) during their physiology and also due to acquired abnormalities. Exosomes have been detected in plasma, serum, urine, synovial fluid, and several other biological fluids. The isolation of exosomes is complex due to their heterogeneity and their physiochemical and biochemical similarities with others EVs. Commonly used strategies for exosome isolation are ultracentrifugation, ultrafiltration, size-exclusion chromatography, polymer centrifugation and microfluidics immunoaffinity capture methodology. All of these methods have their advantages and disadvantages; however, there is currently not a robust, reproducible and standardized method that allows obtaining high purity exosomes (14). Ultracentrifugation-based techniques still remain the gold standard for exosome isolation, despite their limitations (15).

Endosomal budding is the classical procedure proposed for exosome biogenesis, which comprises three main steps: first, an early-sorting endosome (ESE) is formed by endocytosis; second, the endosomal membrane buds inward, generating multiple intraluminal vesicles (ILVs) and, therefore, ESE mature into multivesicular bodies (MVBs). This step is essential in cargo sorting and can occur via two types of pathways: the endosomal-sorting complex required for transport (ESCRT) complex-dependent pathway and the ESCRT complex-independent transport; and third, MVBs fuse with the plasma membrane releasing ILVs, which form exosomes, to the extracellular space (16). Additionally, evidence also supports that exosomes bud from the plasma membrane (13).

In addition to their heterogeneity in size, cellular origin and functional impact, exosomes are widely diverse in content. Exosomes can enclose proteins, RNA, DNA and metabolites, although not all exosomes have a similar abundance of a given cargo. The markers also differ between exosomes, which is based in part on the pathway followed in the biogenesis. The cell of origin and its microenvironment have an impact on the content of the exosomes. However, certain nucleic acids and proteins, compared to the cell of origin, are enriched in exosomes (17).

Exosomal proteins can derive from the membrane, cytosol, nucleus and ECM, and they are distributed both in the membrane and within the lumen of the exosomes. The exosomal proteome helps determine their effects in different types of target. Two sets of exosomes can exert antagonistic effects on their respective receptor cells (13). Regarding the use of proteins as exosomal biomarkers, the levels of tetraspanins CD9, CD63 and CD81, which are used to purify and define exosomes, appear to be heterogeneous, with low expression in exosomes of certain cellular populations. A study suggests that biogenesis-related proteins are ubiquitous in exosomes and that one of them, syntenin-1, could be used for exosome detection due to its high abundance (18). In what concerns exosomal RNA, exosomes are enriched in small noncoding RNAs (ncRNAs), such as microRNAs (miRNAs), small nuclear RNAs and fragmented RNAs, but they also present mRNAs. Exosomal RNA is composed of a skewed subpopulation of cellular RNA and is enriched in determined RNA species related to the cellular RNA profile. Those RNAs can be transferred to other cells and tissues via exosomes, transforming them (19).

## Exosomes and cancer development: a double-edged sword

Exosomes are involved in several diseases, and play an important role in cancer. Exosomes are implicated in tumor initiation, progression, angiogenesis, metastasis, and drug resistance (20). Tumor cells release larger amounts of exosomes than normal cells, with multiple strategies to favoring tumor-promoting exosomes. In fact, certain elements of the exosome biogenesis machinery are oncogenes or tumor suppressors, and aberrant expression of its components can directly modulate the composition, functions and quantity of exosomes (21). Moreover, evidence suggests that exosomes secreted by polarized cells diverge in composition depending on whether they originate from the apical or basolateral side (16).

Tumor progression depends on the cross-talk between cancer cells with each other, with normal cells and immune system. Due to their role in cell-to-cell communication, exosomes have a huge impact on the tumor microenvironment (TME) (20). For example, a study indicates that in NSCLC, tumor-derived exosomes with high expression of one circular RNA (circRNA) called circUSP7 promote CD8^+^ T cell dysfunction and resistance to anti-PD1 (22). The TME designates to the cellular environment surrounding the tumor, comprising the non-cancerous host cells such as immune cells, endothelial cells and fibroblast; and non-cellular components, which englobe the ECM and soluble products, including molecules released by the tumor (23).

As mentioned above, tumor-derived exosomes can influence recipient cells and reprogram their phenotype, promoting carcinogenesis through a myriad of different processes. For example, A549 lung cancer cells exposed to fine particulate matter secrete exosomes with high levels of Wnt3a, and these exosomes stimulate A549 cell proliferation *in vitro* and *in vivo*, via Wnt3a/β-catenin pathway (24). In another study, human bronchial epithelial cells (HBEC) were transformed to a mesenchymal phenotype common in NSCLC. Exosomes derived from the mesenchymal HBECs containing ZEB1 mRNA, a relevant transcription factor in epithelial-mesenchymal transition (EMT), could induce parental HBECs conversion to a mesenchymal and chemoresistant phenotype (25). In colorectal cancer (CRC), miRNA miR-25-3p is transferred via exosomes from CRC cells to endothelial cells, promoting vascular permeability and angiogenesis, which increased CRC metastasis in liver and lung of mice (26). In summary, proteins, RNAs, DNAs and metabolites associated with exosomes can modify the fate of recipient cells, either the cell itself that releases them by autocrine pathway or other cells via paracrine signaling (27).

On the other hand, exosomes can also have antitumor effects. For example, exosomes derived from antigen-presenting cells, such as dendritic cells (DCs), can activate naïve immune cells, and exosomes of pro-inflammatory M1 macrophages have been reported to propagate and induce an immune stimulating microenvironment (28). Even tumor-derived exosomes can activate immune responses, through antigens and/or MHC-peptide complexes that activate T cells through cross-presenting by DCs or direct presentation (27).

Besides, exosomes have become promising clinical tools due to their properties. The therapeutic use of exosomes as vehicles for the delivery of corrective cargo is being actively investigated. This interest is based on exosome stability, permeability and specific cell recognition, coupled with their ability to pass through the blood-brain barrier and minimal immunogenicity (17, 27). Furthermore, mesenchymal stem cells (MSC)-derived exosomes have some unique characteristics and may be therapeutic by themselves, making them one of the best options to develop drug carriers *in vivo* (29). Another application of exosomes is their potential role as biomarkers. As mentioned above, exosomes can be isolated, characterized and detected from liquid biopsy, opening up doors to the use of exosomal RNAs and proteins in the diagnosis, treatment, monitoring and prediction of prognosis of cancer and other diseases (30). For example, differences in exosomal miRNAs are being investigated to establish a set that could be used to discriminate between conditions, such as NSCLC patients and healthy subjects, patients with NSCLC and with SCLC or for the early detection of NSCLC (31–33). It is relevant to emphasize that the use of exosomes in the diagnosis, prognosis, prediction, and monitoring of the disease extends beyond tumor-derived exosomes: it can also encompass those derived from immune cells. For instance, in cases of head and neck cancers, the ratio of tumour/immune-cell-derived exosomes changed during and after therapy, showing a substantial decrease after surgical procedures. This ratio increased during the follow-up period, registering a higher ratio in non-responders compared to responders (34). Given the predominant focus of studies on tumor-derived exosomes, knowledge regarding the potential applications of immune cell-derived exosomes as biomarkers remains limited. Exploring this specific area of study could yield interesting insights in the future. However, there are a few limitations: the high heterogeneity and small size of exosomes make it difficult for their isolation and detection from human body fluids. Therefore, there is no a standardized method for the exosome isolation and the use of exosomal cargo as biomarkers in cancer precision medicine (30).

Synthetizing, the role of exosomes in cancer is complex and can influence tumor progression in both ways. A relevant part of this setting is the effect of exosomes on the interaction between tumor cells and immune system.

## Exosome-mediated communication network between immune and lung cancer cells

Cancer cells develop several mechanisms to escape immune response. Exosomes interact with the immune system components of the TME and exert a direct impact that can suppress or promote immune activity (35). Tumor-derived exosomes can fuse with the surface membrane of the immune cell through receptor-mediated recognition and uptake, releasing their content into the cytoplasm, or deliver signals through interacting with surface molecules, activating downstream cascades that alter recipient cell expression. This interaction contributes to cancer development by silencing antitumor immune responses (36), while immune cell-derived exosomes can have the opposite effect and suppress tumor progression by stimulating immune process such as inflammation (37).

**Figure 1:**
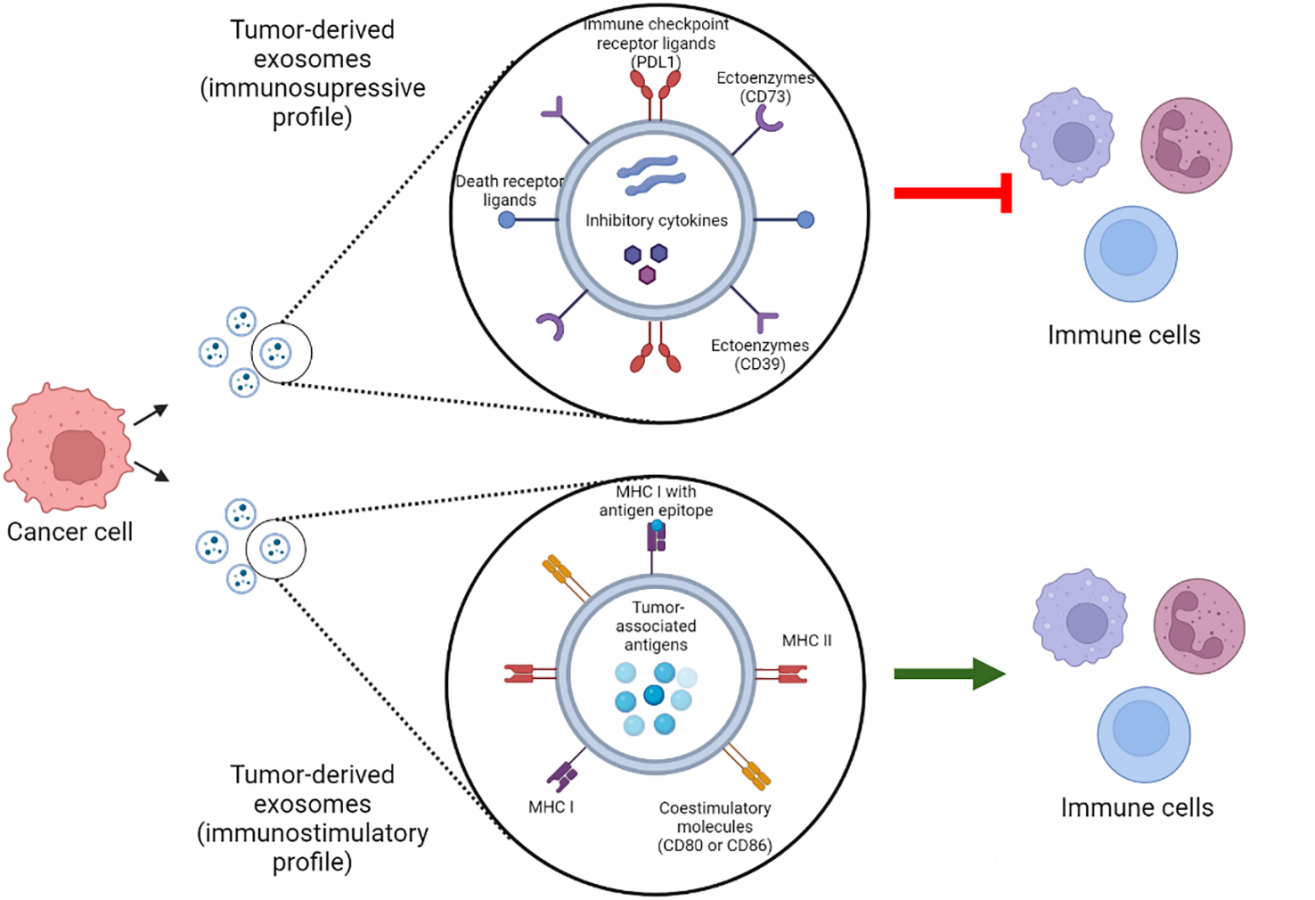
Tumor-derived exosomes molecular profiles. Tumor derived exosomes can contain immunosuppressive and immunostimulatory molecules that modulate their function. Which of these molecules compose the exosome molecular profile and their abundance determine whether the exosome play a tumor suppressing or tumor promoting role. PDL1, programmed death 1 ligand; MHC, Major histocompatibility complex. Created with BioRender.com.

Tumor derived-exosomes can contain either immunosuppressive signaling molecules, such as checkpoint receptor ligands, death receptor ligands, inhibitory cytokines or ectoenzymes; or immunostimulatory molecules such as costimulatory molecules, MHCI/II or tumor associated antigens. Depending on the molecular profile of the exosomes, their function can be tumor suppressing or tumor promoting (36) (**Figure 1**).

**Figure 2:**
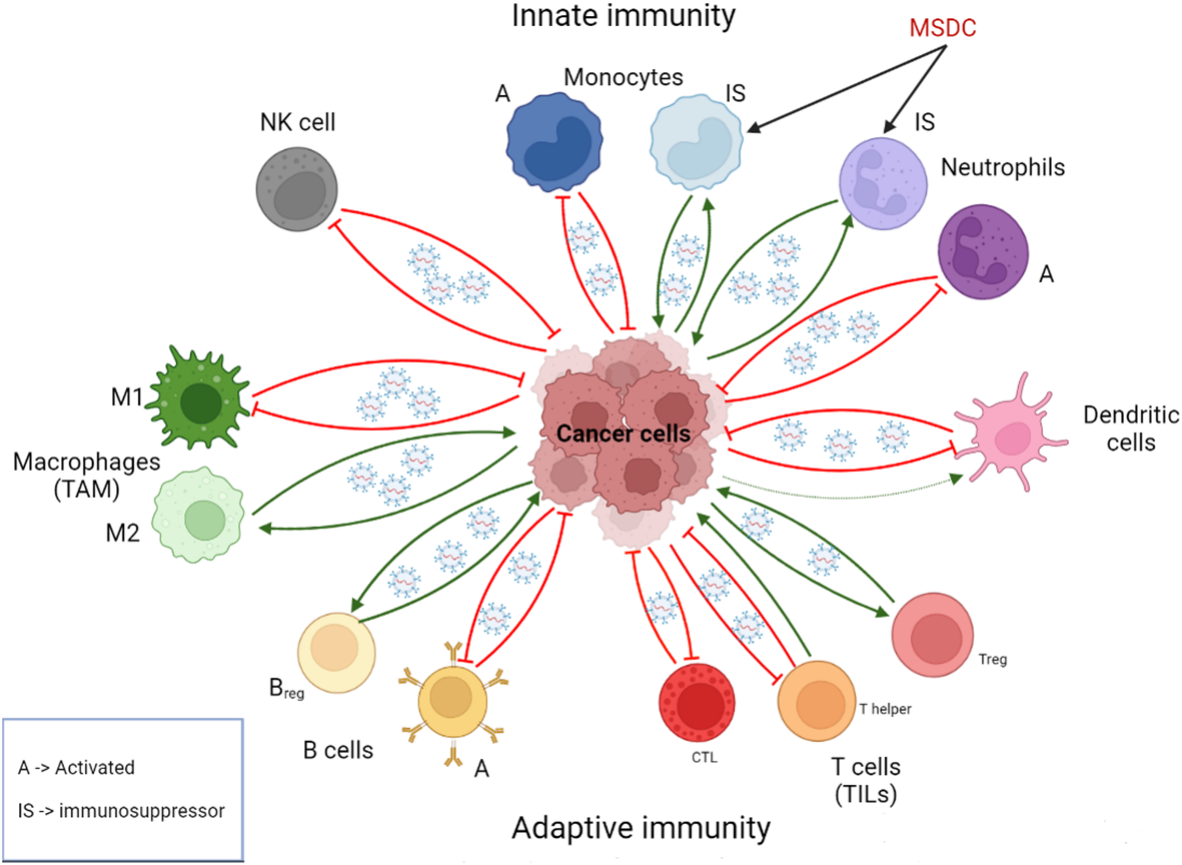
Role of exosomes in the intercommunication between immune cells and cancer cells. Immune cell-derived exosomes can promote or suppress tumor initiation and progression depending on the context and the cell of origin. Usually but not in all cases, exosomes derived from immunosuppressive immune cells are pro-tumorigenic, whereas exosomes derived from activated immune cells are anti-tumorigenic. Cancer cell-derived exosomes can reprogram immune cell, stimulating in general tumor-promoting phenotypes. A, activated; Breg, Regulatory B cells; CTL, CD8^+^ T cells; IS, immunosupressor; MSDC, Myeloid-derived suppressor cell; NK, natural killer; TAM, tumor-associated macrophages; Treg: regulatory T cell; TILs, tumor-infiltrating lymphocytes. Created with BioRender.com.

External factors also participate in the interaction between immune system and carcinogenesis. The lungs are particularly exposed to the environment, including tobacco and pathogenic microorganisms. Smoking and pulmonary infections promote lung cancer complications through chronic inflammation and, therefore, through the infiltration of inflammatory cells that release proinflammatory factors, leading to mutations and the stimulation of tumorigenic process such as metastasis (38). Exosomes play a role in this interaction. For example, macrophages produce IL-6, IL-10 and other proinflammatory cytokines that induce characteristics of stem cells in cancer cells. This communication, which contributes to cell growth, can be established by exosomes in addition to secretory pathways and cell fusion (39). Thus, immune cell-derived exosomes do not invariably suppress tumor progression, resulting in a more complex interaction network. Therefore, there is a wide spectrum of immune cells both innate (macrophages, dendritic cell, neutrophils, among others) and adaptive (B and T cells) that, together with cancer cells, determines whether the immune response is pro- or anti-tumorigenic (40), being a key factor exosomal cargo mediating immune cell-tumor cell communication (**Figure 2**). Concerning immune cell-derived exosomes, their cargo may contain proteins and miRNAs that exert either immunosuppressive or immunostimulatory effects. Often, these molecules exhibit similarities to those previously described for tumor-derived exosomes, including proinflammatory proteins and checkpoint receptor ligands, such as the proinflammatory proteins S100A8 and S100A9 in myeloid-derived suppressor cell (MDSC)-derived exosomes or PDL1 in T cell-derived exosomes (41, 42). Given the diverse range of immune cells, a myriad of molecules could be involved in these processes.

### Innate immune response cells

Innate or nonspecific immunity comprises the cells that respond to multiple antigens with the same mechanism, conforming the first line of defense (43). Some of these cells also fulfill a role in adaptive immunity.

#### Natural killer cells

NK cells are the main effector component of innate immunity due to their ability to autonomously kill target cells, playing a role in tumor immunosuppression. Cancer cells present several mechanisms to avoid elimination mediated by NK cells, leading to a procarcinogenic immune response (44). First, the cytotoxic activity of NK cells extends to EV produced by them, such as exosomes. Therefore, NK cells-derived exosomes show antitumor properties (45). In lung cancer patients, NK cell-derived exosomes carrying miR-30c were significantly downregulated compared to NK cell-derived exosomes from healthy individuals. *In vitro* knockdown of miR-30c was shown to decrease the cytotoxic activity of NK cells against lung cancer cells. Polypeptide N-acetylgalactosaminyltransferase 7 was proposed as target gene of miR-30c and involved in the activation of the PI3K/AKT pathway (46). Therefore, the GALTN7-miR30c axis modulates PI3K/AKT activity, considered as a hallmark of cancer. On the other hand, seen from another point of view, tumor-derived exosomes can also help avoid the elimination of cancer cells mediated by NK cells. Lung cancer-derived exosomes have been proposed to promote NK cell apoptosis by binding of pro-nerve growth factor to the p75 neurotrophin receptor-sortilin complex (47). Previous studies show the presence of sortilin in lung cancer exosomes (48), although there are still no experimental data to support this hypothesis. In addition, therapeutic approach as radiotherapy can promote NSCLC-derived exosomes production and are released of a radiation dose rate-dependent manner, leading to NK cell polarization in peripheral blood mononuclear cells (PBMCs) and immune cell redistribution (49). These cell phenotype changes induced by NSCLC-derived exosomes released by radiotherapy could trigger a more or less effective antitumor immune response.

#### Monocytes

Monocytes are innate immune cells can differentiate into tumor-associated macrophages (TAMs) and DCs. Moreover, they perform other tasks such as phagocytosis, lymphocytes recruitment, promotion of angiogenesis, remodeling of the ECM, and releasing of tumoricidal mediators, thus contributing to pro- and anti-tumoral immunity (50). Tumor derived exosomes have the ability to alter monocytes differentiation, which leads to the formation of immunosuppressive CD14^+^ HLA-DR^lo/neg^ monocytes, characterized for the absence of HLA-DR and costimulatory molecules. CD14^+^ HLA-DR^lo/neg^ monocytes abundance directly correlates with poor clinical outcomes (51).

There is little information about the relationship among lung cancer, exosomes and monocytes. Exosomes derived from pulmonary epithelial cancer cells metabolize leukotriene C4 to leukotriene D4, a pro-inflammatory factor that promotes bronchoconstriction and other effects, and is related to some chronic inflammatory diseases. Monocytes are a major source of leukotriene C4 in the lung TME, which conversion to leukotriene D4 stimulates proliferation and migration of cancer cells (52). In contrast, the relevance of this subset of cells have been described in others cancer types. For example, Pancreatic cancer-derived exosomes promote an immunosuppressive phenotype increasing STAT3 signaling, fostering an incremented expression of arginase and reactive oxygen species production in monocytes (53). Considering their role in other tumors and that high levels of these monocytes influence negatively to the clinical outcome of NSCLC patients administered with a telomerase peptide vaccine (54), it would be important to deepen in the interrelation between exosomes and monocytes in lung cancer.

#### Neutrophils

Neutrophils are a central component of innate immune response and the first responders to infection and inflammation, performing functions such as phagocytosis, degranulation, and release of neutrophil extracellular traps. Within peripheral leukocytes, neutrophils are the most abundant population in humans. Traditionally, depending on their role in cancer, neutrophils have been classified into two types: tumor-restraining N1 population and tumor-promoting N2 population. However, plasticity and heterogeneity of neutrophils may indicate that this binary compartmentalization is obsoleted (55).

Tumor-derived exosomes foster a protumor N2 phenotype in several types of cancer, such as gastric cancer, where exomes carrying HMGB1 induced the N2 phenotype by TLR4/NF-κB signaling (56) or CRC, where CRC stem cell-derived exosomes enhances neutrophils survival through the transfer of triphosphate RNAs that stimulates IL-1β expression (57). Regarding lung cancer, exosomes derived from nicotine-activated N2 neutrophils promote brain metastasis, via a process that implicates exosomal miR-4466: N2 neutrophils with activated STAT3 are recruited into the premetastatic niche and secreted the mentioned miRNA, promoting stemness and a metabolic switching that foster metastasis (58). This shows the relevance of environmental factors, such a smoking, in lung cancer, and how these factors can influence in the content and function of exosomes. Hence, it is essential to consider the smoking habit in studies related to exosomes and the lung.

#### Myeloid-derived suppressor cells

Monocytes and neutrophils with an immunosuppressive phenotype are named MDSCs. Despite they usually are described in pathological conditions, MDSCs also play a role in physiological processes. Based on their myeloid cell lineage origin, MDSC are classified into granulocytic/polymorphonuclear MDSCs (PMN-MDSCs) and monocytic MDSCs (M-MDSCs). In fact, N2 neutrophils terminology describes PMN-MDSCs (59).

Tumor-derived exosomes from diverse types of cancer potentiates MDSCs induction. Lewis lung carcinoma (LLC) cell line derived exosomes stimulate MDSC expansion in a mouse tumor model and in CD14^+^ monocytes *in vitro*, through a miR-21a-dependent mechanism. Exosomal miR-21a downregulates programmed cell death protein 4, which inhibits IL6 production in bone marrow cells (60). Tumor derived exosomes can also promote MDSC differentiation into other immunosuppressive immune cells: exosomal circPTK2/circHIPK3, observed to be enriched in the serum of Kras-related NSCLC patients compared to healthy individuals, especially in metastatic stages, stimulate monocytic MDSC recruitment and their differentiation into alternatively activated (M2) macrophages (61), contributing to favoring pro-tumorigenic processes. On the other hand, MDSCs-derived exosomes also contribute to tumor progression. For example, exosomal miRNA-143-3p derived from PMN-MDSCs downregulates integral membrane protein 2B in lung cancer tissues, activating PI3K/AKT signaling pathway and enhancing cell proliferation (62). Finally, again in lung cancer, PMN-MDSCs have been shown to inhibit NK cell activity through cell-to-cell contact, MDSCs-derived exosomes act as inhibitory mediators of NK cells, and exosomal cargo included several immune-modulatory miRNAs, such as miR-146a-5p, miR-150-5p, miR-378a-3p, miR27a-5p, and miR-218-5p (63).

#### Macrophages

Macrophages are monocyte-derived cells that phagocyte and clear away cells, pathogens and other harmful matter. They also secrete a myriad of immunomodulatory cytokines that can lead to inflammation. Therefore, they participate in innate immunity and adaptive immunity. Macrophages can be polarized into two main phenotypes: classically activated (M1) macrophages, functionally pro-inflammatory; or M2 macrophages, which are anti-inflammatory. Macrophages recruited to the TME become TAMs. M1-like TAMs contribute to restrain the tumor, whereas M2-like TAMs promote cancer development (35).

Lung tumor-derived exosomes can polarize M0 macrophages to an M2 phenotype, altering their transcriptional and bioenergetics signatures. This polarization showed a general increase in oxygen consumption, which is consistent with the bioenergetics state of M2 macrophages (64). Furthermore, hypoxia appears to induce lung cancer to release exosomes enriched in miRNAs like miR-103a, stimulating angiogenesis and blood vessel permeability in the TME. Tumor-exosomal miR-103a reduces PTEN expression, stimulating macrophage polarization to a M2 phenotype by activating PI3K/Akt and STAT3 pathways (65). In another study, the oncogenic lncRNA long intergenic non-coding RNA 00963 promoted exosome-induced M2 macrophage polarization in ADC, by inhibiting the ubiquitination and degradation of Zeb1, a transcriptional factor related to EMT (66).

Furthermore, tumor-exosomal miRNAs, that are transferred to the recipient cell where they bind to their target mRNA, can function also as agonists of TLR receptors in macrophages to stimulate a prometastatic inflammatory response. Using a mice model injected with LLC cells, tumor-exosomal miR-21 and miR-29a interacted with TLR receptors in macrophages, increasing the secretion of IL6 and TNF-α and stimulating the formation of lung multiplicities (67).

On the other hand, exosomes released by macrophages themselves are also relevant, it was found that M2 TAMs secrete exosomes containing miR-155 and miR-196a-5p that can promote EMT, cell migration, cell invasion and cell viability in NSCLC (68). TAM-derived exosomes can also favor EGFR-TKI resistance in NSCLC via activation of AKT, ERK1/2 and STAT3 signaling pathways (69). On the contrary, macrophages themselves and other immune cells, even some diseased cells, can polarize the naïve macrophages via exosomes toward a M1 phenotype (70).

#### Dendritic cells

DCs are specialized antigen-presenting cells that modulate immune signals through cell-cell contacts and cytokines. DCs play a central role in the initiation of antigen-specific immunity and tolerance, presenting antigens to naïve T cells, promoting their activation and effector differentiation. Therefore, DCs are key elements in the antitumor immune response. However, certain inhibitory signals induce in DCs a tolerogenic phenotype, leading to T cell apoptosis and Treg cells generation. TME contains immunosuppressive factors that can inhibit DC infiltration and favor the tolerogenic phenotype, generating dysfunctional DCs (71).

Tumor-derived exosomes can contribute to the formation of tolerogenic DCs, which alter the immune response. In a study with the lung cancer cell lines LLC and A549, lung carcinoma cell-derived exosomes suppress DC costimulatory molecule expression, inflammatory response and promote DCs autophagy via MALAT1, a transcript that stimulates the AKT/mTOR pathway (72). In a xenograft mouse model of EGFR-mutant lung cancer, LLC cells transferred EGFR with the deletion mutation E746-A750 to the surface of the DCs via exosomes. This induction of anergic DCs dampened the proliferation of T cells within the lymph nodes and diminished tumor T cell infiltration (73). Tumor-derived exosomes can also block DC maturation: exosomes derived from LLC cells suppress DC maturation and migration, ultimately reducing T helper 1 (T_H_1) cell differentiation and increasing regulatory T (T_reg_) cell levels. PDL1 may be involved in this process (74). Additionally, exosomes secreted by other cellular types influence DCs function: Treg derived exosomes transferred miR-150-5p and miR-142-3p to DCs, promoting a tolerogenic phenotype by upregulating IL10 and downregulating proinflammatory cytokine IL6 production (75).

On the other hand, tumor-derived exosomes also have been shown to promote DCs maturation and enhance antigen presentation, stimulating tumor specific CTLs response with more potential that tumor cell lysates (76). Dendritic cell-derived exosomes, also known as dexosomes, can be loaded with antigens and present them to naïve DCs potentially eliciting a strong antitumor activity. Hence, tumor-derived exosomes could be used to develop DCs vaccines that foster antitumor immune response (76, 77).

### Adaptive immune response cells

Adaptive or specific immunity is directed against a determined pathogen that has been previously presented. Their main cellular component are T and B lymphocytes (43).

#### B lymphocytes

B cells play a crucial role in the humoral response, producing antibodies that may be secreted or incorporated into the plasma membrane of the cell. In addition to producing immunoglobulins, B cells can influence the functions of other immune cells by releasing cytokines, presenting antigens and providing costimulation. B cells comprehend a functionally heterogeneous population: the balance among the different subsets in the TME may determine whether B cells contribute or suppress immune response (78).

Regulatory B cells (B_reg_) are a subset of B cells that correlate with poor clinical outcome in certain types of cancer. B_reg_ cells produce molecules such as IL10, IL21 or PDL1 and promote T_reg_ cell production, thus inhibiting pro-inflammatory lymphocytes (79). Tumor-derived exosomes enhance B_reg_ proliferation and increase their resistance to apoptosis through mechanism dependent on IL10 and TGF-β, which later inhibit CTLs activities (80). There is a lot of field to be studied, for example, it could be interesting to analyze the effects of B cell populations on the lung TME.

#### T lymphocytes

There are three main subtypes of T lymphocytes: cytotoxic CD8^+^ T cells (CTLs), which are able to directly kill the target cell; CD4^+^ T helper cells, which activate and promote the effector and memory functions of CTLs and modulate the TME; and T_reg_ cells, related to the mechanism of tolerance necessary to avoid autoimmunity. CTLs and T_H_1 cells play a major role in anti-tumor immune responses, while T_reg_ cells contributes to their suppression (81). Lymphocytes infiltrated in the TME configure the tumor-infiltrating lymphocytes (TILs) population. The present of TILs, in general, constitutes a favorable prognostic factor in NSCLC, although the composition of the TILs population determines its contribution to the disease. For example, population enriched in FOXP3^+^ T_reg_ is associated with worse prognosis (82, 83).

CTLs, when activated, can eliminate tumor cells. Hence, their abundance correlates positively with a better clinical response to immunotherapy. However, CTL function can be disrupted by several mechanisms that induce an exhausted phenotype, which cannot respond to the presentation of the antigen. One of them is the aberrant activation of the immune checkpoints: When receptors such as PD1, T cell immunoglobulin and mucin domain-3 protein (Tim-3), T cell immunoglobulin and ITIM domain (TIGIT), lymphocyte-activation gene 3 (Lag-3), or cytotoxic T lymphocyte antigen-4 (CTLA-4), localized on the T cell surface, interact with their ligands, an inhibitory signal is sent to the T cell (84). In lung cancer, exosomes can induce T cell exhaustion, which promotes the expression of PDL1 in cancer cells or PD1 expression in CTLs. This has been observed in lung ADC, where tumor cells upregulate circRNA-002178, which is delivered through exosomes to promote PDL1 and PD1 expression (85). Exosomes can also contain PDL1, allowing them to suppress immune response at distance, not only locally, by systematically inhibiting T cell function in TME (86). Another mechanism consists of inhibiting the secretion of cytokines by CTLs. Exosomal circUSP7 secreted by NSCLC cells inhibits the secretion of IFN-γ, TNF-α, Granzyme-B and Perforin. Moreover, exosomal circUSP7 upregulates Src homology region 2-containing protein tyrosine phosphatase 2, a protein recruited by the PD1/PDL1 interaction that suppress TCR-mediated signaling (22).

The link between T helper cells and lung cancer progression is more complex. The recruitment and expansion of T_H_1 cells promote an effective antitumor immune response. The role of T helper 17 (T_H_17) cells is ambivalent: they can either enhance the activation of immune cells or stimulate cancer development through angiogenesis and immunosuppression processes (35). There are few studies about the relationship between exosomes and T helper cells in lung cancer. Nevertheless, there is evidence that both are associated with cancer. For example, T helper cell-derived EVs, in addition to IL2, foster CTLs activation in a melanoma mouse model, therefore enhancing antitumor immune response (87). On the other hand, tumor-derived exosomes cause the loss of CD69 on the surface of conventional CD4^+^ T cells, leading to their functional decline (88).

T_reg_ cells are a subgroup of CD4^+^ T cells. Their presence in the TME contributes to tumor progression due to their immunosuppressive functions. In a study with *in vitro* and *in vivo* models, the effects of tumor-derived exosomes isolated from LLC cells on lung tumor formation and metastasis were investigated. The lung fibroblast secreted large amounts of CCL1, which activated its specific receptor CCR8 that induced the differentiation of CD4^+^ FOXP3^+^ T_reg_ cells. The number of T_regs_ cells in the lungs increased, promoting the establishment of an immunologically tolerant pre-metastatic niche (89). Moreover, another study showed that tumor-derived exosomes induce GPX3 expression in a subpopulation of alveolar type 2 epithelial cells, inducing high production of IL-10 and ultimately improving T_reg_ cells generation and inhibiting the proliferation of T helper cells (90). These data suggest that cancer cells can recruit T_regs_ cells to induce tumor-tolerance via exosomes. Tumor-derived exosomes can also upregulate the biological activities of T_reg_ cells. For example, tumor-derived exosomes foster the expression of CD39 in several types of cancer, an ATP-hydrolase that increases the production of the immunosuppressive factor adenosine (79).

There is a fourth subtype of T lymphocyte of interest that has been associated with lung cancer via exosomes: the CD3^+^CD4^−^CD8^−^ double-negative T (DNT) cells. These cells express PD1 and the TNF-related apoptosis-inducing ligand (TRAIL) in high quantities, among others. In a study, DNT cells present in malignant pleural effusions (MPE) derived from lung cancer patients expressed PD1 and TRAIL at higher levels. Whereas DNT cells from healthy donors could kill lung cancer cells, this activity was reduced by MPE: MPE supernatant-derived exosomes, exhibiting immune checkpoint molecules like CEACAM1 and PDL1, have been observed to inhibit the cytotoxicity of DNT cells, at least partially, through CEACAM1/TIM3 and PDL1/PD1 pathways. The blockage of Tim-3 and PD1 rescued the cytotoxic potential in DNT cells (91).

## Role of exosomes in lung cancer immunotherapy

**Figure 3:**
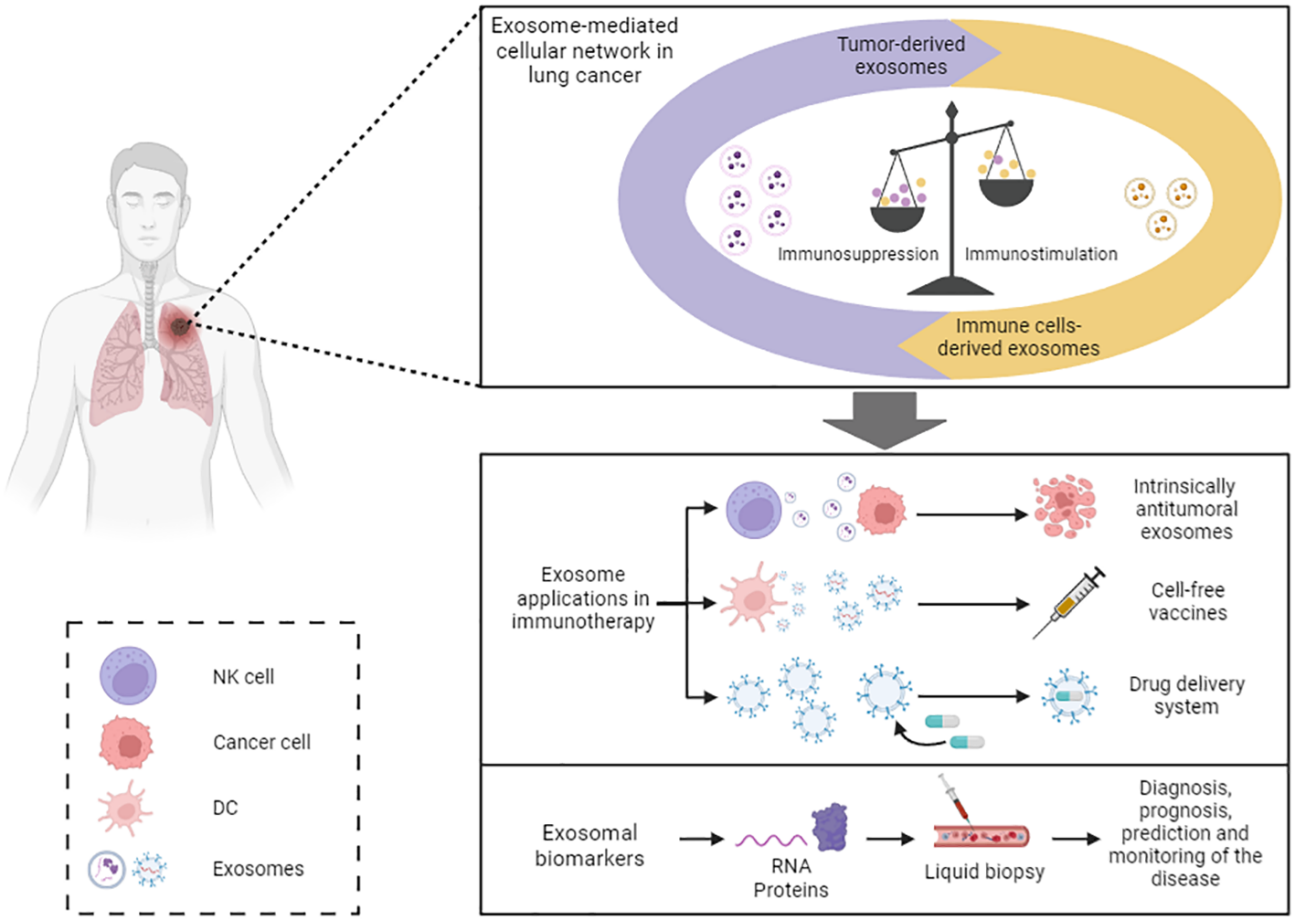
Exosome-based immunotherapy. Exosome-based immunotherapy. Exosomes offer diverse therapeutic applications: exosomes derived from specific cell types, like NK cells and MSC, exhibit inherent antitumor properties that could be harnessed; exosomes derived from DCs and tumor cells could be utilized as cell-free vaccines; and exosomes may serve as effective drug delivery systems. Moreover, exosomal cargo can be employed as biomarkers of the disease. NK, natural killer; DC, dendritic cell.

Throughout this review, several mechanisms by which exosomes can enhance the immune response have been explained. Therefore, exosomes have the potential to be used as therapeutic tools to induce antitumor immunity (**Figure 3**) (35). For example, the antitumor properties of NK cells-derived exosomes could be employed as a personalized cancer therapy. They have the shared advantage with NK cells of not requiring antigen presentation for their effector activity, but offer higher stability, modification versatility, and lower immunogenicity compared to their cells of origin (45). It has been observed that EVs derived from NK cells stimulated with cytokines IL-15 and IL-21 exhibit enhanced cytotoxic activity against cancer cells (92). Hence, the use of NK cells-derived exosomes could be an interesting objective to pursue in immunotherapy.

As mentioned above, MSC-derived exosomes also possess intrinsic therapeutic properties. There are several studies that relate the biomedical applications of MSC with their released components, opening up the possibility of cell-free therapies in regenerative medicine that overcome the side effects associated with stem cell transplantation (93). For example, MSC-derived EV have been used in diverse *in vivo* models of inflammatory lung diseases, exhibiting therapeutic effects similar to those of their cells of origin (94). The role of MSC-derived exosomes in cancer is more complex, with contradictory functions that can either promote angiogenesis and tumor development or suppress tumor growth and progression (93). Considering the multiple cell types that release exosomes with the potential to stimulate immune response and antitumor immunity, and that these cells often can play opposite roles depending on the TME, we believe that the use of exosomes from immunoresponsive cancer patients to stimulate the immune response in patients with an immunosuppressive TME could be an interesting area of study.

The use of exosomes as delivery systems could be leveraged for immunotherapy. For example, engineered exosomes could enable the reprogramming of TAMs, targeting M2-like TAMs and inducing their polarization to an M1-like phenotype: exosomes with the clustered regularly interspaced short palindromic repeats interference (CRISPRi) internally engineered and a TAM specific peptide expressed onto the membrane were produced. These exosomes silenced PI-3 kinase gamma and promoted TAMs polarization to M1, which reactivated tumor immunity (95). In another study, they used M1 macrophages exosomes transfected with NF-κB p50 siRNA and miR-511-3p to foster M2-like TAM reprogramming into a M1 phenotype, inhibiting tumor growth and stimulating anti-tumor immune response (96). Therefore, the modification of the exosomal content and its targeted delivery may be a good mechanism for cell repolarization.

Another potential application of exosomes in immunotherapy is their use as a cell-free vaccine. Antigen-loaded dexosomes can start an antitumor immune response and can be obtained from monocyte-derived DCs isolated by peripheral blood leukapheresis procedure (97). Dexosomes contain different antigen-presenting molecules, such as MHC I/II proteins and costimulatory factors like CD86, that can trigger CTLs and CD4^+^ T cells activation and proliferation (98). For example, exosomes derived from DCs primed with A549 cell lysates induced T cell proliferation and allogeneic PBMCs mediated cytotoxicity against A549 lung cancer cells *in vitro* (99). The use of dexosomes as cell-free anticancer vaccines has been studied in several phase I and phase II clinical trials, demonstrating their ability to trigger both innate and adaptive immune responses, through T cells and NK cells, respectively. However, despite their excellent tolerability and safety, dexosomes showed limited therapeutic benefit (100–103). Clinical trials related to exosome-based cell-free vaccines for lung cancer are listed in **Table 1**. The results obtained with dexosomes vaccines improve in combination with other therapy regimes. For instance, when combined with metronomic oral low-dose cyclophosphamide, CTLs priming against tumor increased in mice, although metronomic oral low-dose cyclophosphamide-dexosome combination chemotherapy in humans, which has been implemented in the phase II trial, appears to require the presence of genuine adjuvants (97).

**Table 1:** Clinical trials employing exosomes related to immune response.

ClinicalTrials.gov identifier	Recruitment status	Phase	Cancer type	Exosome clinical use	Intervention/treatment
NCT01159288	Completed	II	NSCLC	Dexosome Vaccine	Dex2
NCT04427475	Recruiting	NA	NSCLC	Biomarker	Plasma exosomes
NCT02869685	Unknown	NA	NSCLC	Biomarker	Plasma exosomes
NCT02921854	Completed	NA	NSCLC	Biomarker	Serum exosomes

NA, Not applicable; Dex, tumor antigen-loaded dendritic cell-derived exosomes.

Tumor cell-derived exosomes can also stimulate antitumor immunity and be used as cell-free vaccines. In a study, a nanovesicle was designed by engineering tumor cell-derived exosomes with the fibroblast activation protein-α (FAP) gene, with the purpose of serve as a tumor vaccine and to overcome the immunosuppressive characteristics of TME. FAP is overexpressed in cancer-associated fibroblasts, which constitute the most abundant stromal cells within the TME. This vaccine induced a strong CTL immune response against tumor cells and FAP^+^CAF and remodeled the immunosuppressive TME in lung, colon, breast, and melanoma cancer models (104).

The potential use of exosomes and their cargo as biomarkers is another field that can be exploited in cancer immunotherapy. Several exosomes-dependent mechanisms are related to anti-tumor immunity resistance. The ICIs targeting PD1 and PDL1, despite showing a good clinical response in a variety of cancer types, fail to ameliorate the disease in a significant number of patients. This resistance to therapy could be caused by exosome-mediated systemic immunosuppression. As discussed above, two main mechanisms of exosome-mediated resistance are endogenous tumor exosomal PDL1 and PDL1 expression induced by tumor-derived exosomes (105). Exosomes-based liquid biopsies could be used to predict whether a patient will respond to the treatment and even monitor their response over time. For example, PDL1 overexpression is necessary for pembrolizumab treatment, an anti-PD1 antibody that blocks the immune checkpoint, and mutational burden is linked with a major clinical benefit in pembrolizumab treatment. Exosome-derived biomarkers may contribute to determining these factors before initiating the therapy, without the need to obtain a tissue biopsy, and enable the selection of the most appropriate treatment for each patient (106). Several studies have focused on the search for exosomes-derived biomarkers that can predict and monitor cancer therapy immunoresistance or immunosensitivity (107, 108). In fact, most clinical trials related to exosomes and lung cancer are associated with their use as biomarkers (**Table 1**).

## Future perspective and conclusions

There is a plethora of research that support the relevance of exosomes in cell communication and in the interrelationship between processes such as cancer and immunity. In lung cancer, as in other types of cancer, exosomes can promote or prevent the initiation and development of tumors by regulating the immune response, among other mechanisms. Although the vast majority of the bibliography has been focused on the role of tumor-derived exosomes, exosomes released by immune cells and other cell types composing the TME have an important role in the disease and should be taken into consideration to better understand the origin and evolution of the disease. In the same way, the intercommunication between tumor cells and other cells types has been extensively studied, but it could be interesting to analyze the interaction between the different cell subsets that compose the TME, as it could have indirect effects on the tumor.

Besides, due to lung exposition to the environment, external factors play a fundamental role in the disease. Tobacco consumption and infections are related to pro-carcinogenic immune responses; therefore, it could be relevant to study their effects on immune cells and exosome release, not only their role in cell malignization. In addition to the interaction between external factors and endogenous exosomes, the role of exogenous exosomes, such as dietary exosomes, should be considered. Edible exosomes may be a potential immunotherapeutic tool against cancer (109, 110), although exosomes derived from determined foods have been associated with cancer promotion (109).

As discussed in this review, exosomes have enormous potential as immunotherapy tools due to their characteristics and role in the disease. Exosomes could provide a solution to the challenges in cancer drug delivery, because of their stability in circulation, biocompatibility, and low immunogenicity. Additionally, surface engineering helps to locally concentrate exosomes at the compromised site, reducing possible side effects (111). However, further studies are necessary for the application of these therapies at the clinical level. Meanwhile, exosome-based vaccines have been used in clinical trials and could be a safer alternative to cell vaccines, although the antitumor immune responses achieved have been limited. The use of engineered exosome-like nanovesicles could contribute to improved clinical outcomes. The most extended application of exosomes in clinical trials is their use as biomarkers: liquid biopsies can serve for prediction, diagnosis, prognosis, and real-time tumor monitoring of the disease. The use of exosomes and their cargo as biomarkers would facilitate precision medicine in lung cancer and oncology in general, helping to select the best treatment and reject other alternatives in the early stages of the disease (12). However, to establish this in the clinical routine, several obstacles that must be overcome. Regarding exosome isolation, there is no standardized method for high yield and high-purity separation. Exosome similarities with other EVs, heterogeneity, and low isolation efficiency complicate reliable molecular characterization for clinical practice. The establishment of a standardized high-throughput isolation method, alongside future technologies, such as single exosome detection and analysis technology, could help to overcome these issues (30).

In summary, exosomes play a critical role in lung cancer and immunity, incredibly complex and sometimes ambiguous: a cell can release pro-tumorigenic and anti-tumorigenic exosomes, depending on the cell context and expression patterns. Considering that exosomes are a key component of cell communication, the analysis of the interaction among the different components of the TME is crucial to fully understand their relevance in the disease, although a vast number of studies focus on the effects of individual exosomal molecules. Due to their biological role and characteristics, exosomes are interesting tools and targets for immunotherapy. Despite being in its beginnings, it is an evolving field with great potential.

## Author contributions

AC-P: Investigation, Writing – original draft, Writing – review & editing. SM-P: Investigation, Writing – original draft, Writing – review & editing, Conceptualization, Supervision.

## Glossary

**Table:**

SCLC	Small-cell carcinoma
NSCLC	Non-small-cell carcinoma
ADC	Adenocarcinoma
SqCC	Squamous cell carcinoma
LCLC	Large-cell carcinoma
PD1	Programmed death 1
PDL1	Programmed death 1 ligand
ICI	Immune checkpoint inhibitors
EV	Extracellular vesicles
ESE	Early-sorting endosome
ILVs	Intraluminal vesicles
MVBs	Multivesicular bodies
ESCRT	Endosomal-sorting complex-independent transport
ncRNAs	Small noncoding RNAs (ncRNAs)
miRNAs	microRNAs
TME	Tumor microenvironment
circRNA	circular RNA
ECM	extracellular matrix
HBEC	Human bronchial epithelial cells
EMT	Epithelial-mesenchymal transition
CRC	colorectal cancer
DCs	dendritic cells
MSC	Mesenchymal stem cells
NK	Natural Killer
PBMCs	Peripheral blood mononuclear cells
TAMs	Tumor-associated macrophages
MDSCs	Myeloid-derived suppressor cells
PMN-MDSCs	Polymorphonuclear MDCs
M-MDSCs	Monocytic MDSCs
LLC	Lewis Lung carcinoma
M2 macrophages	Alternatively activated macrophages
M1 macrophages	Classically activated macrophages
T_H_1 cell	T helper 1 cell
T_reg_ cell	Regulatory T cell
B_reg_ cell	Regulatory B cell
CTLs	Cytotoxic CD8^+^ T cells
TILs	Tumor-infiltrating lymphocytes
Tim-3	mucin domain-3 protein
TIGIT	T cell immunoglobulin and ITIM domain
Lag-3	Lymphocyte-activation gene 3
CTLA4	Cytotoxic T lymphocyte antigen-4
T_H_17 cell	T helper 17 cell
DNT cell	Double-negative T cells
TRAIL	TNF-related apoptosis-inducing ligand
MPE	malignant pleural effusions
CRISPRi	clustered regularly interspaced short palindromic repeats interference
FAP	Fibroblast activation protein- α.
